# Elevated risk of attention deficit hyperactivity disorder (ADHD) in Japanese children with higher genetic susceptibility to ADHD with a birth weight under 2000 g

**DOI:** 10.1186/s12916-021-02093-3

**Published:** 2021-09-24

**Authors:** Md Shafiur Rahman, Nagahide Takahashi, Toshiki Iwabuchi, Tomoko Nishimura, Taeko Harada, Akemi Okumura, Nori Takei, Yoko Nomura, Kenji J. Tsuchiya

**Affiliations:** 1 Research Centre for Child Mental Development, Hamamatsu University School of Medicine, 1-20-1 Handayama, Higashi-ku, Hamamatsu, 431-3192 Japan; 2 United Graduate School of Child Development, Osaka University, Kanazawa University, Hamamatsu University School of Medicine, Chiba University and University of Fukui, Hamamatsu, Japan; 3 Department of Child and Adolescent Psychiatry, Nagoya University Graduate School of Medicine, Nagoya, Japan; 4 Institute of Psychiatry, Psychology & Neuroscience, King’s College London, London, UK; 5 Queens College and Graduate Center, City University of New York, New York, NY USA

**Keywords:** Birth weight, Polygenic risk, ADHD, Inattention, Hyperactivity, Cohort study

## Abstract

**Background:**

Both genetic and pre- and perinatal factors, including birth weight, have been implicated in the onset of attention deficit hyperactivity disorder (ADHD) traits among children. This study aimed to elucidate to what extent the genetic risk of ADHD moderates the association between birth weight and ADHD traits among Japanese children.

**Methods:**

We conducted a longitudinal birth cohort study (Hamamatsu Birth Cohort for Mother and Children Study) to investigate the association of genetic risk for ADHD and low birth weight with ADHD traits among Japanese children. Out of 1258 children**,** we included 796 who completed follow-ups at 8 to 9 years of age. Birth weight was categorized as <2000 g, 2000–2499 g, and ≥2500 g. Polygenic risk score for ADHD was generated using the summary data of a large-scale genome-wide association study. The Rating Scale IV (ADHD-RS) assessed ADHD traits (inattention and hyperactivity/impulsivity) based on parental reports. Following previous studies, sex, birth order of the child, gestational age at birth, mother’s age at delivery, educational attainment, pre-pregnancy body mass index, pre-pregnancy or during pregnancy smoking status, alcohol consumption during pregnancy, father’s age, education, and annual family income were considered as covariates. Multivariable negative binomial regression was applied to evaluate the association between birth weight and ADHD traits, while adjusting for potential covariates. The interaction term between birth weight categories and binary polygenic risk was added to the model.

**Results:**

Birth weight of 2000–2499 g was not associated with ADHD traits. Birth weight under 2000 g was significantly associated with both inattention and hyperactivity. When accounting for higher and lower genetic risk for ADHD, only those with higher genetic risk and birth weight < 2000 g were associated with inattention (rate ratio [RR] 1.56, 95% CI 1.07–2.27) and hyperactivity (RR 1.87, 95% CI 1.14–3.06)**.**

**Conclusions:**

Birth weight under 2000 g, together with the genetic risk of ADHD, contributes to higher levels of ADHD traits among Japanese children aged 8 to 9 years. The suggested association between low birth weight and ADHD is confined to children with a genetic susceptibility to ADHD, indicating the relevance of genetic-environmental interactions in the etiology.

**Supplementary Information:**

The online version contains supplementary material available at 10.1186/s12916-021-02093-3.

## Key points

- **Questions:** Does genetic risk moderate the association between birth weight and ADHD traits among Japanese children?
- **Findings:** Children with birth weight under 2000 g and higher genetic risk of ADHD showed increased severity of ADHD traits at age 8 to 9 years, compared to those with either normal birth weight or lower genetic risk.
- **Meaning:** Japanese children born with birth weight less than 2000 g are more vulnerable to their genetic susceptibility to ADHD. Additional attention is needed to minimize the severity of ADHD traits during childhood.

## Background

Attention deficit hyperactivity disorder (ADHD) is one of the most prevalent neurodevelopmental disorders [1, 2], accounting for 1.14 million disability-adjusted life years globally in 2017 [3]. ADHD can be classified as predominantly inattentive, predominantly hyperactive/impulsive, or a combination of both and usually appears during early childhood and continues to manifest throughout the life course [1, 4]. ADHD affects functioning, both in school and daily activities [5], and increases the social and economic burden [6, 7]. In addition, children with ADHD symptoms are often bullied, neglected, and ignored by their peers and others in society due to their inattentive and restless behavioral phenotypes [8]. Although the etiology of ADHD is complex and multifactorial, both pre- and perinatal conditions and genetic risk factors have been implicated in the development of ADHD symptoms among children [9–11]. Specifically, low birth weight [12] is the most consistent environmental factor identified in the literature to affect neurodevelopment and the onset of ADHD in childhood [13].

Accumulating evidence suggests that low birth weight is an early biomarker for newborn infants’ vulnerability to experiencing altered neurodevelopment and developmental disorders [14, 15]. A recent meta-analysis reported that children born with very low birth weight have a higher risk of developing ADHD during childhood [14]. Although several previous studies have investigated the association between birth weight and ADHD traits, the results remain inconclusive [16–24]. For instance, some studies found a negative relationship between birth weight and ADHD symptoms [17–19, 22, 23], while others failed to confirm the association [25, 26]. Moreover, the majority of previous studies have explored the link between ADHD and the extremely low end of the birth weight distribution [14, 27], that is, either very low birth weight (≤1500 g) or extremely low birth weight (≤1000 g), or a low birth weight classified as under 2500 g [18, 21]. A birth weight of 2500 g is the standard cutoff set by the World Health Organization (WHO) to define low birth weight and is generally considered to indicate high-risk newborns. However, the cut-off point for low birth weight remains controversial. Given that Asian children are generally lighter than their Western counterparts [28], the limit of 2500 g is inadequate for defining low birth weight. Recently, a multi-national study suggested a birth weight of around 2000 g as the threshold for defining low birth weight among Asians [29]. Notably, the mean birth weight in Japan has declined significantly in the last three decades (1980–2010) [30]. Thus, the prevalence of low birth weight, particularly birth weights between 1500 to 2499 g, has increased significantly [30]. Therefore, it is of paramount importance to investigate whether intermediate birth weight categories, such as 1500 to 1999g and 2000 to 2499g, are associated with ADHD symptoms among Japanese children.

Besides the birth weight implications for ADHD, it is well known that ADHD is heritable (70–80%) [31, 32]. A previous meta-analysis found significant associations between ADHD and eight candidate variants connected with six genes [33, 34]. In addition, a recent genome-wide association study (GWAS) identified 304 genetic variants in 12 loci that were associated with ADHD [35]. Furthermore, several studies found a significant association between genetic risk, specifically polygenic risk scores (PRS), and ADHD traits [36–38].

The influence of some individual candidate genes and several prenatal factors on ADHD traits is known to some extent. While previous studies have highlighted the importance of gene-environmental interactions in the etiology of ADHD [39, 40], the evidence of this relationship remains limited [40–42]. Thus, the interactions of genetic liability, measured using PRS, with prenatal factors such as birth weight could elucidate the etiology of ADHD [43]. Understanding the mechanistic pathway between genetic risks and non-genetic risks, such as low birth weight, adjusted for lighter Asian infants, may help identify children at greater risk for ADHD diagnosis later in childhood. This may subsequently reduce the long-term sequelae associated with elevated ADHD traits through timely implementation of behavioral and pharmacological interventions [44]. However, studies on the interactions between birth weight and the genetic risk of ADHD on the symptoms of ADHD in childhood are limited. Therefore, this study aimed to investigate the association of low birth weight and genetic risk for ADHD with the presence of ADHD traits in children. We used a longitudinal birth cohort of the general population in Japan. We hypothesized that a higher polygenic risk of ADHD, together with a lower birth weight, would be associated with higher severity of ADHD traits among Japanese children aged 8 to 9 years.

## Methods

### Study cohort

This study used data from the Hamamatsu Birth Cohort for Mother and Child (HBC) Study, which is an ongoing prospective birth cohort study designed to investigate the neurodevelopmental trajectories of children in the general population. The children in the cohort were born in Hamamatsu City, Japan, between November 2007 and March 2011. The details of the HBC study are presented elsewhere [45]. In the HBC study, out of 1258 children, 826 were successfully followed up until 8 to 9 years. We excluded twins (*n*=28) and children with Down syndrome (*n*=2) from this study. Ultimately, 796 children and their mothers were included in this study.

### Exposures

#### Birth weight categories

Birth weight below 1500 g and 2500 g are recognized as very low birth weight and low birth weight, respectively. However, in this study, we had only three children (out of 796) whose birth weight was below 1500 g. Moreover, a recent multi-national study has highlighted the need for redefining low birth weight with a threshold of birth weight of around 2000 g among Asian children [29]. Thus, we classified birth weight into three categories: normal birth weight (≥2500 g), intermediate birth weight (2000–2499 g), and birth weight <2000 g.

#### Polygenic risk score for ADHD

The genetic risk for ADHD in the HBC study was assessed using PRS. PRS represents the approximate genetic liability for a disease or disorder using the number of risk alleles from a set of single-nucleotide polymorphisms (SNPs) [46]. The detailed estimation procedure of the PRS and quality controls of SNPs in the HBC study were described in our previous study [47]. Trained clinicians collected buccal swabs (DNA samples) from children during the follow-up surveys conducted either at age 32 months, 40 months, 4.5 years, or 6 years. In this study, out of 796 children, a total of 137 children were excluded from the PRS analysis because genomic DNA was unavailable due to a low call rate (<97%) or a refusal or reluctance of buccal swab collection. A Japonica array was used for SNP genotyping [48] and BEAGLE 5.0 of phase 3 of the 1000 genome project was used as the reference panel for Japanese populations in the genotyping imputation [49]. Next, the PRS was generated in PRSice-2 software using a previous GWAS study for ADHD, conducted by the Lundbeck Foundation Initiative for Integrative Psychiatric Research, as the discovery cohort [50]. Although the ADHD-PRS was estimated at several *p*-value thresholds, we considered the best fit PRS for this study. The ADHD-PRS for the included 659 samples had a bell-shaped distribution with a mean of 0.00124 and median of 0.00123 (Supplementary Figure 1). The score in the middle of the distribution (a PRS value of 0) corresponds to the average risk for developing ADHD. The score on the right tail of the distribution indicates a relatively higher genetic risk of ADHD than the average, and the left tail represents a lower risk. When investigated the distribution of genetic risk across children with different birth weight categories, we observed that children were almost equally distributed to higher or lower genetic risk group in each birth weight category (see Additional file 1: Figure S1).

### Outcome: ADHD traits

The main outcomes were the behavioral traits of ADHD, which comprise inattention and hyperactivity domains. To quantify these domains, we used the ADHD Rating Scale IV (ADHD-RS) based on parental reports to assess the severity of ADHD traits at ages 8 to 9 years. The ADHD-RS comprises 18 items covering the spectrum of the inattention (9 items) and hyperactivity/impulsivity domains (9 items) [51]. Responses are rated on a 4-point Likert scale ranging from 0 (“never” or “rarely”) to 3 (“very often”) [51].

### Covariates

Following previous studies [52, 53], the sex, birth order of the child, gestational age at birth, mother’s age at delivery, educational attainment, pre-pregnancy body mass index (BMI), pre-pregnancy or during pregnancy smoking status, alcohol consumption during pregnancy, father’s age at birth, education, and annual family income (at birth) were considered as confounders and were included in the model.

### Statistical analysis

Frequency distribution and univariate statistics were used to describe the characteristics of children and their mothers. The data were not normally distributed as indicated by a Shapiro-Wilk test (*p*<0.001 for all three outcomes related to ADHD-RS total, inattention, and hyperactivity scores). Therefore, negative binomial regression models were employed to investigate the association between the exposure and outcome variables. The choice of negative binomial models was determined by considering overdispersion that was present for both the Poisson and zero-inflated Poisson models. The negative binomial regression model provided rate ratios (RR) along with 95% confidence intervals (CIs), which indicates the number of times that the ADHD score is higher (when RR>1) or lower (RR<1) than the reference group. In the case of continuous exposure, an RR value higher than 1 indicates a higher severity associated with a one-unit increase in the exposure, and vice versa. Model 1 was adjusted for variations in survey time only. Model 2 was additionally adjusted for gender, birth order of the child, maternal age at delivery, education, pre-pregnancy BMI, smoking status, and alcohol intake to control for maternal and infant-related factors. Model 3 was further adjusted to include the father’s age at birth, education, and annual family income to control for paternal factors. We found significant interaction effects between birth weight categories and ADHD-PRS. To facilitate interpretation, we classified the PRS into a binary variable using the median value as the threshold. Therefore, any PRS above the median was defined as “having higher genetic risk than average” and PRS values below the median were considered as “having lower genetic risk than average”. We also considered the interaction of birth weight categories and binary polygenic risk variable in the model to estimate the RR for each combination of birth weight category and binary polygenic risk variable. Clustering was allowed for with the Huber-White sandwich estimator in all the included models, as some children included in this study were born to the same mother. Furthermore, background characteristics of children included in the analysis and those excluded from the analysis were compared. The Little’s test for Missing Completely at random (MCAR) was used to test whether missingness of ADHD trait scores and ADHD-PRS were completely at random [54], while an extension of the Little’s MCAR test, with several combinations of auxiliary variables as covariates into the test, was employed to test the covariate-dependent missingness [55]. Then we performed multiple imputation of ADHD-PRS for children with missing information, assuming missing completely at random, to evaluate the potential impact of missing observations (17.2%) on the association between birth weight and ADHD traits. In addition, we performed sensitivity analyses by considering missing ADHD-PRS to the end of the spectrum, i.e., recoding all missing ADHD-PRS as high risk or low risk. Statistical analyses were performed using Stata MP version 16.1.

## Results

### Characteristics of participants

Table 1 shows the demographic characteristics of the study participants. A total of 796 children (49.2% females; 50.8% males) aged 8 to 9 years were included in this study. Among them, 6.4% were born preterm (before the gestational age of 37 weeks). The mean (±SD) birth weight and gestational age at birth were 2949.3 g (440.1) and 38.9 weeks (1.52), respectively. Additionally, 87.3% of children had a normal birth weight, 10.3% had a birth weight between 2000 to 2499 g, and 2.4% had a birth weight under 2000 g. The median scores for ADHD combined traits, as well as inattention and hyperactivity traits, were significantly higher among children who had a birth weight < 2000 g (see Additional file 2: Table S1). These scores were lowest among children with birth weights between 2000 and 2499 g (see Additional files 3 and 4: Figures S2-S3).

**Table 1:** Background characteristics of study participants (*N*=796)

Characteristics	*n*	%	Mean or median	SD or IQR
*Children’s characteristics*				
Birth order, *n* (%)				
First	401	50.4		
Second or higher	395	49.6		
Gender, *n* (%)				
Girls	392	49.2		
Boys	404	50.8		
Gestational age at birth in week, mean (SD)			38.9	1.52
Preterm birth, *n* (%)				
Yes	51	6.4		
No	745	93.6		
Birth weight (g), mean (SD)			2949.3	440.1
<2000 g	19	2.4		
2000–2499 g	82	10.3		
≥2500 g	695	87.3		
ADHD-PRS, mean (SD)			0.0022	1.01
ADHD-RS total score, median (IQR)			5^a^	1–10^b^
Inattention, median (IQR)			3^a^	1–7^b^
Hyperactivity, median (IQR)			1^a^	0-3^b^
*Parent’s characteristics*				
Mother’s age at delivery, *n* (%)				
<35 years	567	71.2		
≥35 years	229	28.8		
Maternal educational attainment, *n* (%)				
≤12 years	246	30.9		
>12 years	550	69.1		
Maternal pre-pregnancy BMI, *n* (%)				
Underweight (<18.5)	170	21.3		
Normal weight (18.5-24.9)	541	68.0		
Overweight (≥25.0)	85	10.7		
Pre-pregnancy or during pregnancy smoking history, *n* (%)				
Yes	155	19.5		
No	641	80.5		
Alcohol consumption during pregnancy, *n* (%)				
Yes	110	13.8		
No	686	86.2		
Father’s age at birth				
<25 years	45	5.6		
25–34 years	456	57.3		
≥35 years	295	37.1		
Father’s education at birth				
≤12 years	59	7.4		
>12 years	737	92.6		
Household annual income at birth in JPY				
≤3 million	36	4.5		
3–8 million	604	75.9		
≥8 million	156	19.6		

Note: ^a^Values present median; ^b^values present interquartile range; *ADHD-RS*, attention deficit/hyperactivity disorder rating scale; *BMI*, body mass index; *IQR*, interquartile range; *PRS*, polygenic risk score; *SD*, standard deviation; *JPY*, Japanese yen

### Independent effects of birth weight and ADHD

In the negative binomial models, birth weight, when considered as continuous, was not significantly associated with ADHD scores (see Additional files 5: Table S2). However, when birth weight was considered as categorical, we found that birth weight < 2000 g was significantly associated with higher ADHD trait scores (RR 1.60, 95% CI 1.16–2.22) (Table 2). Similar findings were observed for both inattention and hyperactivity domains. Birth weight < 2000 g was significantly associated with higher scores of both inattention (RR 1.50, 95% CI 1.10–2.06) and hyperactivity traits (RR 1.82, 95% CI 1.17–2.83) compared to normal birth weight (≥2500 g) (model 1). After adjusting for potential covariates in model 3, the results remained statistically significant for both inattention (RR 1.49, 95% CI 1.15–1.94) and hyperactivity traits (RR 1.78, 95% CI 1.19–2.67).

**Table 2:** Association of birth weight categories with ADHD scores among Japanese children at age 8–9 years (*N*=796)

Birth weight	Rate ratio (95% confidence intervals)		
	ADHD total score	Inattention score	Hyperactivity score
**Model 1**^†^			
Normal birth weight (ref.)	1.00	1.00	1.00
Birth weight 2000–2499 g	0.88 (0.68–1.13)	0.90 (0.70–1.15)	0.83 (0.60–1.15)
Birth weight < 2000 g	1.60 (1.16–2.22)**	1.50 (1.10–2.06)*	1.82 (1.17–2.83)**
**Model 2**^‡^			
Normal birth weight (ref.)	1.00		
Birth weight 2000–2499 g	1.00 (0.77–1.30)	1.00 (0.77–1.29)	0.99 (0.70–1.40)
Birth weight < 2000 g	1.60 (1.21–2.12)**	1.51 (1.17–1.95)**	1.85 (1.23–2.77)**
**Model 3**^§^			
Normal birth weight (ref.)	1.00		
Birth weight 2000–2499 g	1.00 (0.77–1.29)	1.00 (0.77–1.29)	0.99 (0.71–1.39)
Birth weight < 2000 g	1.57 (1.19–2.09)**	1.49 (1.15–1.94)**	1.78 (1.19–2.67)**

Note: Normal birth weight was defined as birth weight ≥ 2500 g; *ref*., reference category; values in bold show statistical significance; **p<0.01; *p<0.05

^†^Model 1 was adjusted for variations in survey time only

^‡^Model 2 additionally adjusted for gender of child, parity, maternal age, education, pre-pregnancy body mass index, pre-pregnancy smoking status, and alcohol intake

^§^Model 3 additionally adjusted for father’s age at birth and household annual income

### Interaction of genetic risk of ADHD and birth weight on ADHD traits

We found a significant interaction effect of birth weight categories and ADHD-PRS on ADHD scores (Wald chi-squared test, *p*<0.05 for ADHD total score) (see Additional files 6: Table S3). This provided evidence of the moderating effects of genetic risk on the association between birth weight and ADHD traits. Thus, we included the interaction of birth weight categories and binary polygenic risk variables in the model.

**Fig. 1:**
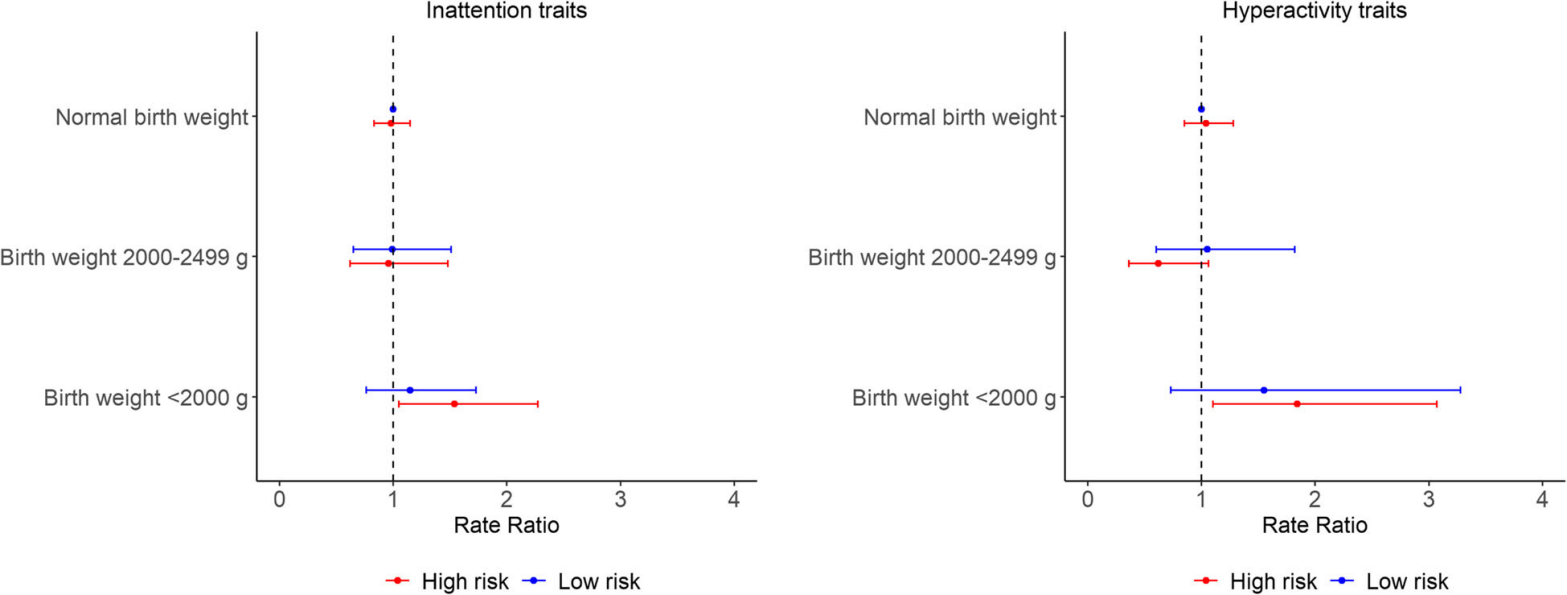
Multivariable-adjusted rate ratios (RR) and 95% confidence intervals (CI) for the association of birth weight categories and genetic risk with inattention and hyperactivity scores among Japanese children at age 8–9 years. Note: Normal birth weight was defined as birth weight ≥ 2500 g and considered as the reference category. Models were adjusted for gender of child, parity, maternal age, education, pre-pregnancy body mass index, pre-pregnancy smoking status, alcohol intake, variations in survey time, father’s age at birth and household annual income

Higher genetic risk of ADHD and birth weight < 2000 g were associated with higher severity of ADHD traits (RR 1.82, 95% CI 1.10–3.01) compared to those born with normal birth weight or had lower genetic risk (Table 3). Similar findings were observed for both inattention and hyperactivity traits (Table 4 and Fig. 1).

**Table 3:** Association of birth weight categories and genetic risk with ADHD total score among Japanese children at age 8–9 years (*N*=659) (a total of 137 children with missing information on PRS was excluded from this analysis)

Birth weight and genetic risk of ADHD	Rate ratio (95% confidence intervals)		
	Model 1^†^	Model 2^‡^	Model 3^§^
Normal birth weight			
Low risk (ref.)	1.00	1.00	1.00
High risk	0.98 (0.82–1.16)	0.97 (0.83–1.14)	0.98 (0.83–1.15)
Birth weight 2000–2499 g			
Low risk	0.88 (0.56–1.37)	1.04 (0.68–1.61)	1.04 (0.68–1.61)
High risk	0.85 (0.55–1.31)	0.88 (0.56–1.38)	0.87 (0.56–1.37)
Birth weight <2000 g			
Low risk	1.32 (0.83–2.09)	1.32 (0.80–2.19)	1.30 (0.79–2.16)
High risk	**1.82 (1.10**–3.01)*	**1.68 (1.17**–2.39)**	**1.64 (1.14**–2.35)**

Note: Normal birth weight was defined as birth weight ≥ 2500 g; *ref*., reference category; values in bold show statistical significance; **p<0.01; *p<0.05

^†^Model 1 was adjusted for variations in survey time only

^‡^Model 2 additionally adjusted for gender of child, parity, maternal age, education, pre-pregnancy body mass index, pre-pregnancy smoking status, and alcohol intake

^§^Model 3 additionally adjusted for father’s age at birth and household annual income

**Table 4:** Association of birth weight categories and genetic risk with inattention and hyperactivity scores among Japanese children at age 8–9 years (*N*=659) (a total of 137 children with missing information on PRS was excluded from this analysis)

Birth weight and genetic risk of ADHD	Rate ratio (95% confidence intervals)		
	Model 1^†^	Model 2^‡^	Model 3^§^
**Inattention symptoms**			
Normal birth weight			
Low risk (ref.)	1.00	1.00	1.00
High risk	0.96 (0.81–1.13)	0.95 (0.81–1.12)	0.96 (0.82–1.13)
Birth weight 2000–2499 g			
Low risk	0.89 (0.58–1.35)	1.02 (0.67–1.56)	1.02 (0.67–1.56)
High risk	0.93 (0.62–1.39)	0.96 (0.62–1.50)	0.97 (0.62–1.50)
Birth weight <2000 g			
Low risk	1.21 (0.82–1.77)	1.22 (0.80–1.84)	1.19 (0.78–1.81)
High risk	**1.70 (1.02**–2.83)*	**1.59 (1.09**–2.31)*	**1.56 (1.07**–2.27)*
**Hyperactivity symptoms**			
Normal birth weight			
Low risk (ref.)	1.00	1.00	1.00
High risk	1.02 (0.81–1.29)	1.04 (0.84–1.27)	1.03 (0.84–1.27)
Birth weight 2000–2499 g			
Low risk	0.86 (0.50–1.49)	1.10 (0.62–1.93)	1.10 (0.63–1.91)
High risk	0.68 (0.36–1.28)	0.64 (0.37–1.11)	0.63 (0.37–1.08)
Birth weight <2000 g			
Low risk	1.56 (0.73–3.31)	1.65 (0.75–3.65)	1.64 (0.77–3.48)
High risk	**2.10 (1.10**–3.98)*	**1.94 (1.19**–3.17)**	**1.87 (1.14**–3.06)*

Note: Normal birth weight was defined as birth weight ≥ 2500 g; *ref.*, reference category; values in bold show statistical significance; **p<0.01; *p<0.05

^†^Model 1 was adjusted for variations in survey time only

^‡^Model 2 additionally adjusted for gender of child, parity, maternal age, education, pre-pregnancy body mass index, pre-pregnancy smoking status, and alcohol intake

^§^Model 3 additionally adjusted for father’s age at birth and household annual income

In model 1, birth weight under 2000 g and higher genetic risk of ADHD were significantly associated with higher scores of inattention (RR 1.70, 95% CI 1.02–2.83) and hyperactivity (RR 2.10, 95% CI 1.10–3.98). These results remain statistically significant after additionally adjusting for potential confounders in model 3. The corresponding RRs were 1.56 (95% CI 1.07–2.27) for inattention traits and 1.87 (95% CI 1.14–3.06) for hyperactivity traits (Table 4).

### Attrition

We compared background characteristics of children included in the analysis and those excluded due to missing information on ADHD trait scores and ADHD-PRS. The proportion of missing ADHD trait scores or ADHD-PRS was slightly higher among children of younger mother, less educated, and smoker mother (see Additional files 7: Table S4). However, the Little’s test confirmed that missingness of ADHD trait scores and ADHD-PRS were completely at random as well as missingness was not covariate-dependent (see Additional files 8: Table S5). We performed multiple imputation of ADHD-PRS for children with missing information on ADHD-PRS and the results remained virtually the same (*N*=796) (see Additional files 9 and 10: Tables S6-S7). The effect sizes remain almost same and significant in the sensitivity analyses where we considered all missing PRS as high risk or low risk (see Additional files 11 and 12: Tables S8-S9).

## Discussion

Using a prospective birth cohort study, we found that birth weight under 2000 g was significantly associated with an increased likelihood of having higher ADHD trait scores among Japanese children aged 8 to 9 years. Furthermore, such increased risks were confined but significant for children with relatively higher polygenic risk of ADHD and birth weight under 2000 g. This confirms our hypothesis that reduced birth weight (<2000 g), together with higher genetic susceptibility for ADHD, are associated with an elevated risk of developing ADHD traits during childhood. To our knowledge, this is the first epidemiological study to evaluate the joint contribution of birth weight and polygenic risk of ADHD on the domains of inattention and hyperactivity/impulsivity.

Consistent with previous studies [14, 56–58], we found that children with birth weight < 2000 g had approximately 50% higher ADHD trait scores than those born with normal birth weight. This finding is also consistent with previous studies conducted among participants who were clinically diagnosed with ADHD [21, 52, 59]. The effect size for the association remained materially unchanged even after adjusting for parental factors and other potential confounders, suggesting an independent association between birth weight and ADHD traits. Notably, most previous studies considered 1000 g, 1500 g, or 2500 g as the threshold for extremely low, very low, or low birth weight categories, respectively. However, our study focused on intermediate low birth weight categories (i.e., <2000 g; 2000 to 2499 g) and observed a higher risk of ADHD traits for birth weight under 2000 g. This highlights the need for adopting lower cut-off points to define low birth weight regarding ADHD risk. This finding is congruent with previous studies that hypothesized that low birth weight, as an early life adversity (e.g., impaired nourishment in utero), is associated with neurodevelopmental difficulties [60]. Although birth weight < 1500 g is classified as very low in clinical practice, there were only three children in this category in our study. Therefore, we could not evaluate the association between very low birth weight and ADHD traits. Nevertheless, our findings on the higher severity of ADHD traits and birth weight under 2000 g have clinical importance. This is because the percentages of children born with birth weight between 1500 and 1999 g or 2000 and 2499 g are much larger than children born with extremely low or very low birth weight, particularly in Japan [30]. Thus, our study expands on previous research by evaluating the risk estimates for different categories of birth weight, and by adjusting for a wide range of potential covariates. In addition, this study provides evidence of the independent association between low birth weight and ADHD traits among children. It further emphasizes that the neurodevelopmental progress of children with low birth weight under 2000 g should be monitored closely, as these children are at heightened risk of developing ADHD traits in early childhood. Regarding the two domains of ADHD, we found that children whose birth weight was less than 2000 g had 1.49 times higher scores of inattention traits and 1.78 times higher hyperactivity scores. This is consistent with a previous study conducted among British and Brazilian children that observed higher risks of attention difficulties for birth weights under 2500 g [61]. Furthermore, although we found no significant difference between the RRs of inattention and hyperactivity scores for children with birth weights under 2000 g, we found that the risk of hyperactivity was slightly higher than that of inattention (RR 1.78 vs. 1.49). To date, no study has considered the direct comparison of the effect size for hyperactivity traits. Therefore, further studies are necessary to confirm this finding.

By including an interaction term of polygenic risk and birth weight categories to the multivariable models, the risk-conferring effect of low birth weight under 2000 g was found in children with higher genetic risk of ADHD, but not in children of lower genetic risk. This effect highlights that genetic risk could moderate the association between birth weight and ADHD traits. These results were anticipated, as a previous GWAS conducted among English children aged 7 years found a positive association between PRS and inattentive traits, hyperactive/impulsive traits, and overall ADHD traits [36]. In addition, another study conducted among Dutch children found a significant association between ADHD-PRS and clinical ADHD [37]. Unfortunately, we could not compare these effect sizes with previous research, as none of the prior studies evaluated the joint effect of low birth weight and genetic vulnerability on ADHD traits.

Notably, the risk was confined to those born under 2000 g in our Japanese sample, while previous studies from the USA indicated that the risk was observed among children born under 2500 g [62]. This contrast may indicate the relevance of racial differences in maternal and child body weight to the study variables. However, since we adjusted for maternal BMI, and the limited sample size did not allow us to calibrate the effect of small differences in birth weight, future studies with larger sample sizes are warranted to address this as a global public health issue.

### Biological mechanisms

The precise biological mechanisms underlying the association between birth weight and ADHD traits remain uncertain; however, several hypotheses have been proposed by previous studies. It may be attributed to the inadequate supply of oxygen and necessary nutrient transport from the mother’s blood to the fetus, known as prenatal ischemia-hypoxia (PIH), which restricts fetal growth and alters placental development, causing low birth weight [63]. PIH causes several functional problems and neuropsychiatric disorders, including the symptoms of ADHD, through altered brain development of the fetus and increased susceptibility to neurodevelopmental problems [63, 64]. In addition, the immune system increases the release of circulating pro-inflammatory cytokines in response to PIH, resulting in systemic inflammation, which further leads to impaired neurodevelopment [65]. Another possibility is the role of hypothalamic-pituitary-adrenal (HPA) axis dysregulation, which is the main component of the stress response and regulation system. This is common among infants born prematurely or born with low birth weight due to the insufficient supply of nutrients to the fetus [66]. It has been suggested that the disturbances of the HPA axis and resulting fetal exposure to excess glucocorticoid levels may influence developmental pathways, leading to impaired brain development, behavioral impairments, and psychiatric disorders, including ADHD [67, 68]. Further experimental studies are needed to confirm the causal relationships between low birth weight and ADHD traits.

The underlying factors of the interaction of genetic risk for ADHD and low birth weight on ADHD traits remains unclear. Therefore, few hypotheses have been suggested to explain this interaction. The complex blend of numerous common genetic variants, such as dopaminergic, noradrenergic, and serotoninergic genes, and rare genetic variants are likely to be implicated in the pathophysiology of ADHD [33, 69, 70]. Higher genetic susceptibility to ADHD may moderate the association between reduced birth weight and ADHD through impaired inflammatory response [65] and/or dysfunctions of the HPA axis. Specifically, variations in cytokine genes (IL16 and S100B) may moderate the association between birth weight and the severity of ADHD symptoms [71]. In addition, polymorphism in the glucocorticoid receptor gene (NR3C1) moderates the relationship between stress responses and the severity of ADHD symptoms [66, 72]. This may partially explain the elevated risk of ADHD among children with higher genetic risk for ADHD and reduced birth weight. Previous studies also revealed that the complex interaction of reduced birth weight with both common and rare genetic variants associated with ADHD further leads to several neurobiological changes such as dysfunctions in the brain’s default network, cognitive control systems, and the amygdala-frontal or reward circuit [69]. Such neurobiological changes can be further implicated in several neuropsychological impairments, including among individuals with ADHD symptoms [69].

### Limitations and strengths

Our study had several limitations. First, our study had a relatively small sample size, particularly due to the exclusion of participants without genomic DNA collection. The generalizability of the finding is limited to this population until it can be replicated. However, the participants included in the HBC study comprised a representative sample of the Japanese population. Second, our study lacked diagnostic assessment of ADHD. The ADHD traits were measured based on parental reports on their children using ADHD-RS, which may be subject to social desirability bias. However, the ADHD-RS is a widely used, valid, and reliable scale to assess the severity of ADHD symptoms [73]. Third, participants with ADHD-PRS values higher than the median were considered to have a higher genetic risk for ADHD, since PRS has been standardized in our studied sample. Thus, caution should be taken when generalizing these results, as higher genetic risk in this study indicates a higher risk than the average population. Finally, this study was conducted among Japanese children; thus, the findings may not be generalized to other races or ethnic groups as the genetic background is quite variable.

Despite these limitations, our study has several strengths. The major strengths of this study include the prospective design (birth cohort), lower attrition rate, and representativeness of the sample, making the results of this study applicable to the general Japanese populations. Moreover, this is the first study to investigate the association between birth weight and ADHD traits in childhood while accounting for the genetic risks of developing ADHD. As neurodevelopmental disorders are genetically complex to understand, the inclusion of a single gene marker in the model may not be useful. Since the PRS elucidates the genetic risk through the prediction of complex genetic phenotypes, we accounted for both common and rare genetic risk variants of each participant through the inclusion of PRS in this study [74].

### Clinical implications

Taken together, our findings highlight the importance of vigilant monitoring of children born with birth weight < 2000 g, especially for those with genetic susceptibility to ADHD. Our findings have several clinical implications. First, it would help clinicians, based on birth weight information, to decide whether a child is at a greater biological risk of developing ADHD symptoms later on. Subsequently, the genetic risk for ADHD should be assessed for children with reduced birth weight to identify whether their biological risk may be exacerbated by their higher genetic loading for clinical ADHD. Such information would also support clinicians in the early detection of other co-occurring neuropsychiatric diseases, including autism spectrum disorder [37], anxiety [75], and depression [75, 76].

## Conclusions

In conclusion, birth weight < 2000 g is significantly associated with an increase in the severity of inattention, hyperactivity, and combined ADHD traits among Japanese children aged 8 to 9 years. The presence of higher genetic risk for ADHD among children with reduced birth weight further elevates such risk. The introduction of effective interventions aimed at reducing the incidence of low birth weight, especially among children whose parents have known ADHD symptoms and diagnoses, may play a crucial role in reducing the risk of developing ADHD.

## Supplementary Information

**Additional File 1.** Figure S1 - Distribution of polygenic risk score for ADHD in HBC study.

**Additional File 2.** Table S1 - Background characteristics of study participants according to birth weight categories.

**Additional File 3.** Figure S2 - Distribution of ADHD total scores between birth weight categories.

**Additional File 4.** Figure S3 - Distribution of scores on inattention traits and hyperactivity traits between birth weight categories.

**Additional File 5.** Table S2 - Association of birth weight (continuous) and polygenic risk with ADHD scores among Japanese children at age 8-9 years.

**Additional File 6.** Table S3 - Independent and interaction effects of birth weight categories and polygenic risk score for ADHD with ADHD total score among Japanese children at age 8-9 years.

**Additional File 7.** Table S4 - Comparison of background characteristics of children included in the analysis and those excluded from the analysis due missing information on ADHD-RS or PRS.

**Additional File 8.** Table S5 - Little’s test for Missing Completely at random and test for covariate-dependent missingness.

**Additional File 9.** Table S6 - Association of birth weight categories and genetic risk with ADHD total score among Japanese children at age 8-9 years after multiple imputation of missing polygenic risk score for 137 children.

**Additional File 10.** Table S7 - Association of birth weight categories and genetic risk with inattention and hyperactivity scores among Japanese children at age 8-9 years after multiple imputation of missing polygenic risk score for 137 children.

**Additional File 11.** Table S8 - Sensitivity analysis for the association of birth weight categories and genetic risk with ADHD total score among Japanese children at age 8-9 years after recoding missing PRS to the end of the spectrum.

**Additional File 12.** Table S9 - Sensitivity analysis for the association of birth weight categories and genetic risk with inattention and hyperactivity scores among Japanese children at age 8-9 years after recoding missing PRS to the end of the spectrum.

## Data Availability

Data are not publicly available but can be used for onsite analysis on a reasonable request to the corresponding author (Prof. Kenji J. Tsuchiya).

## Abbreviations

ADHD: Attention deficit hyperactivity disorder
CI: Confidence interval
GWAS: Genome-wide association study
HBC: Hamamatsu Birth Cohort for Mother and Child
HPA: Hypothalamic-pituitary-adrenal
PIH: Prenatal ischemia-hypoxia
PRS: Polygenic risk scores
RR: Rate ratio
SNPs: Single-nucleotide polymorphisms
WHO: World Health Organization
