# Supplementary figures and images for: Elevated risk of attention deficit hyperactivity disorder (ADHD) in Japanese children with higher genetic susceptibility to ADHD with a birth weight under 2000 g
Source: BMC Med. 2021 Sep 24;19:229. doi: 10.1186/s12916-021-02093-3 (PMC8461893; doi:10.1186/s12916-021-02093-3)

**Additional File 1: Figure S1** - Distribution of polygenic risk score for ADHD in HBC study


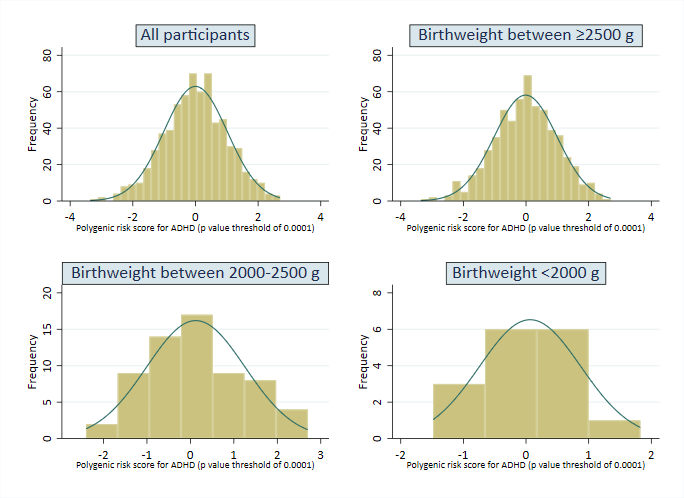

Supplement: Supplementary file 1 — Additional File 1. Figure S1 - Distribution of polygenic risk score for ADHD in HBC study. [file 12916_2021_2093_MOESM1_ESM.docx]
