# Supplementary material for: Elevated risk of attention deficit hyperactivity disorder (ADHD) in Japanese children with higher genetic susceptibility to ADHD with a birth weight under 2000 g
Source: BMC Med. 2021 Sep 24;19:229. doi: 10.1186/s12916-021-02093-3 (PMC8461893; doi:10.1186/s12916-021-02093-3)
Supplement: Supplementary file 2 — Additional File 2. Table S1 - Background characteristics of study participants according to birth weight categories. [file 12916_2021_2093_MOESM2_ESM.docx]

**Additional File 2: Table S1** - Background characteristics of study participants according to birth weight categories (N= 796)

| **Characteristics** | **Total** | **Birth weight** | | | |
| --- | --- | --- | --- | --- | --- |
|  |  | **<2000 g** | **2000-2499 g** | **≥2500 g** | **p-value** |
| *Children’s characteristics* |  |  |  |  |  |
| Birth order, n (%) |  |  |  |  |  |
| First-born | 401 (50.4%) | 10 (52.6%) | 40 (48.8%) | 351 (50.5%) | 0.602 |
| Gender, n (%) |  |  |  |  |  |
| Female | 392 (49.3%) | 9 (47.4%) | 51 (62.2%) | 332 (47.8%) | 0.047 |
| Gestational age at birth in week, mean (SD) | 38.9 (1.5%) | 34.4 (2.4%) | 37.5 (1.5%) | 39.3 (1.5%) | 0.000^†^ |
| Preterm birth n (%) | 51 (6.4%) | 15 (76.0%) | 25 (30.5%) | 11 (1.5%) | 0.000 |
| PRS score for ADHD*, mean (SD) | 0.002 (1.01) | 0.07 (0. 81) | 0.12 (1.13) | -0.01 (1.00) | 0.574^†^ |
| Genetic risk for ADHD*, n(%) |  |  |  |  |  |
| Low risk | 328 (49.8%) | 7 (43.7%) | 30 (47.6%) | 291 (50.2%) | 0.824 |
| High risk | 331 (50.2%) | 9 (56.2%) | 33 (52.4%) | 289 (49.8%) |  |
| ADHD-RS total score, median (IQR) | 5 (1-10) | 10 (4-15) | 4 (1-8) | 5 (1-10) | 0.008^‡^ |
| Inattention, median (IQR) | 3 (1-7) | 7 (3-9) | 3 (1-6) | 3 (1-7) | 0.014^‡^ |
| Hyperactivity, median (IQR) | 1 (0-3) | 3 (1-6) | 1 (0-3) | 1 (0-3) | 0.01^‡^ |
| *Parent’s characteristics* |  |  |  |  |  |
| Mother’s age at delivery, n (%) |  |  |  |  |  |
| <35 years | 567 (71.2%) | 12(63.5%) | 57 (69.5%) | 498 (71.7%) | 0.676 |
| Maternal educational attainment, n (%) |  |  |  |  |  |
| ≤12 years | 246 (30.9%) | 8 (42.1%) | 20 (24.4%) | 218 (31.4%) | 0.245 |
| Maternal pre-pregnancy BMI, n (%) |  |  |  |  |  |
| Underweight (<18.5) | 170 (21.3%) | 5 (26.3%) | 24 (29.3%) | 141 (20.3%) | 0.228 |
| Normal weight (18.5 – 24.9) | 541 (68.0%) | 13 (68.4%) | 47 (57.3%) | 481 (69.2%) |  |
| Overweight (≥25.0) | 85 (10.7%) | 1 (5.3%) | 11 (13.4%) | 73 (10.5%) |  |
| Pre-pregnancy or during pregnancy smoking history, n (%) |  |  |  |  |  |
| Yes | 155 (19.5%) | 4 (21.1%) | 19 (23.2%) | 132 (19.0%) | 0.655 |
| Alcohol consumption during pregnancy, n (%) |  |  |  |  |  |
| Yes | 110 (13.8%) | 1 (5.3%) | 7 (8.5%) | 102 (14.7%) | 0.172 |
| Father’s age at birth |  |  |  |  |  |
| ≥35 years | 295 (37.1%) | 8 (42.1%) | 30 (36.6%) | 257 (36.9%) | 0.844 |
| Father’s education at birth |  |  |  |  |  |
| ≤12 years | 59 (7.4%) | 2 (10.5%) | 10 (12.2%) | 47 (6.8%) | 0.180 |

Note: ADHD, attention deficit/hyperactivity disorder; BMI, body mass index; IQR, interquartile range; PRS, polygenic risk score; SD, standard deviation; Unless stated, the p-values were obtained from Chi-square tests;

^*^ADHD-PRS was missing for 137 children.

^†^One-way ANOVA was performed to compare the mean across birth weight categories;

^‡^Kruskal-Wallis H test was performed to compare the median of three categories of birth weight.
