# Supplementary material for: Elevated risk of attention deficit hyperactivity disorder (ADHD) in Japanese children with higher genetic susceptibility to ADHD with a birth weight under 2000 g
Source: BMC Med. 2021 Sep 24;19:229. doi: 10.1186/s12916-021-02093-3 (PMC8461893; doi:10.1186/s12916-021-02093-3)
Supplement: Supplementary file 3 — Additional File 3. Figure S2 - Distribution of ADHD total scores between birth weight categories. [file 12916_2021_2093_MOESM3_ESM.docx]

**Additional File 3: Figure S2** - Distribution of ADHD total scores between birth weight categories


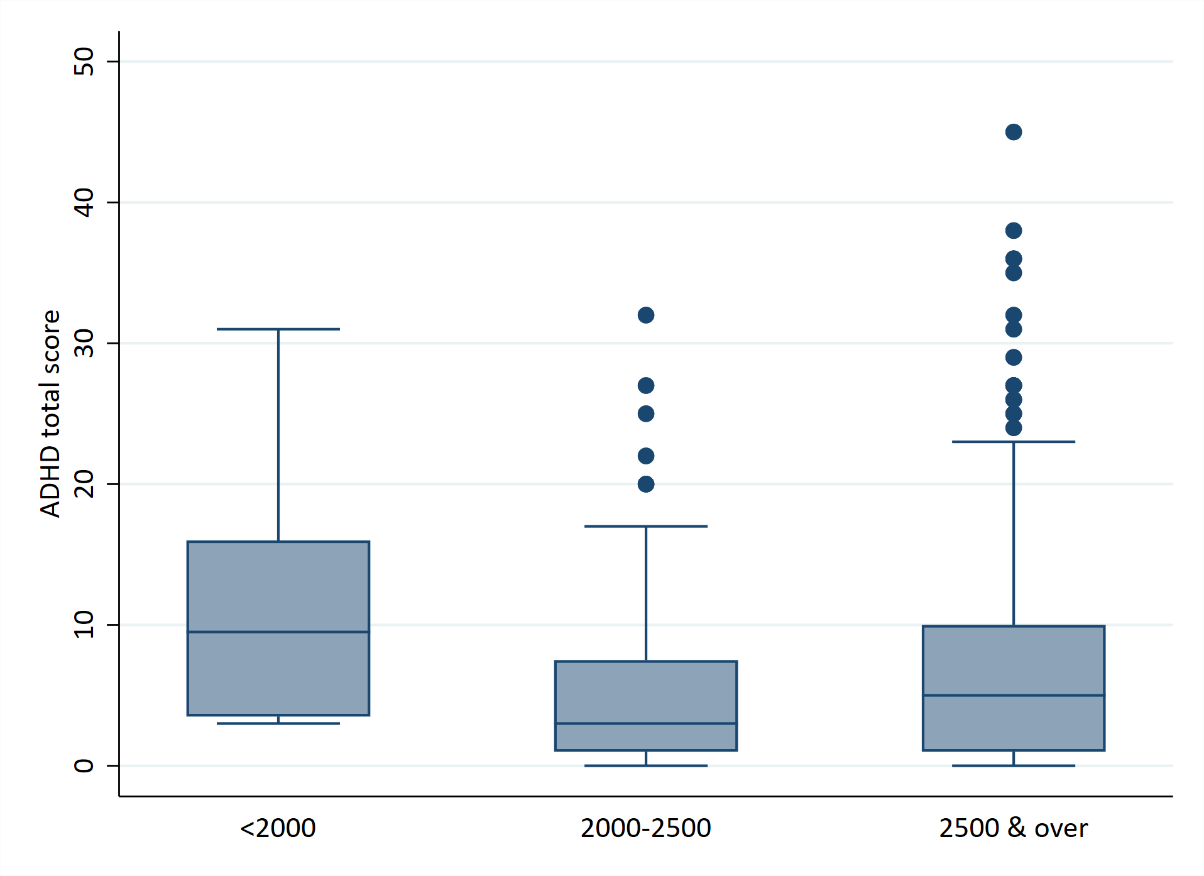


Note: The width of each box represents the interquartile range (IQR) and the solid line within each box shows the median score within the birth weight category. Difference in the median scores across birth weight categories were significant based on Kruskal-Wallis H test (p=0.007).
