# Supplementary material for: Elevated risk of attention deficit hyperactivity disorder (ADHD) in Japanese children with higher genetic susceptibility to ADHD with a birth weight under 2000 g
Source: BMC Med. 2021 Sep 24;19:229. doi: 10.1186/s12916-021-02093-3 (PMC8461893; doi:10.1186/s12916-021-02093-3)
Supplement: Supplementary file 5 — Additional File 5. Table S2 - Association of birth weight (continuous) and polygenic risk with ADHD scores among Japanese children at age 8-9 years. [file 12916_2021_2093_MOESM5_ESM.docx]

**Additional File 5: Table S2** - Association of birth weight (continuous) and polygenic risk with ADHD scores among Japanese children at age 8-9 years (N=659)

| **Characteristics** | **Rate Ratio (95% Confidence Interval)**^†^ | | |
| --- | --- | --- | --- |
|  | **ADHD total score** | **Inattention score** | **Hyperactivity score** |
| Birth weight (continuous) in gram | 0.9999  (0.9998-1.0002) | 0.9999  (0.9998-1.0001) | 0.9999  (0.9997-1.0002) |
| Polygenic risk score (continuous) | 1.03  (0.95-1.11) | 1.02  (0.94-1.0025) | 1.04  (0.93-1.16) |

Note: ^†^Models were adjusted for variations in survey time.
