# Supplementary material for: Elevated risk of attention deficit hyperactivity disorder (ADHD) in Japanese children with higher genetic susceptibility to ADHD with a birth weight under 2000 g
Source: BMC Med. 2021 Sep 24;19:229. doi: 10.1186/s12916-021-02093-3 (PMC8461893; doi:10.1186/s12916-021-02093-3)
Supplement: Supplementary file 6 — Additional File 6. Table S3 - Independent and interaction effects of birth weight categories and polygenic risk score for ADHD with ADHD total score among Japanese children at age 8-9 years. [file 12916_2021_2093_MOESM6_ESM.docx]

**Additional File 6: Table S3** - Independent and interaction effects of birth weight categories and polygenic risk score for ADHD with ADHD total score among Japanese children at age 8-9 years (N=659)

| **Birth weight & genetic risk of ADHD** | **Rate Ratio (95% Confidence Interval)**^†^ |
| --- | --- |
| Birth weight categories |  |
| Normal birth weight (ref.) | 1.00 |
| Birth weight: 2000-2499 g | 0.96 (0.71-1.31) |
| Birth weight <2000 g | 1.43 (1.08-1.89) |
| Polygenic risk score | 1.02 (0.94-1.10) |
| Birth weight categories × Polygenic risk score |  |
| Normal birth weight (ref.) | - |
| Birth weight: 2000-2499 g | 0.98 (0.73-1.32) |
| Birth weight <2000 g | **1.36 (1.06-1.75)**** |

Note: Normal birth weight was defined as birth weight ≥ 2500 g; ref., reference category; Values in bold show statistical significance; ** p<0.01; * p<0.05;

^†^Model was adjusted for variations in survey time, gender of child, parity, maternal age, education, pre-pregnancy body mass index, pre-pregnancy smoking status, alcohol intake, father’s age at birth, and household annual income.
