# Supplementary material for: Elevated risk of attention deficit hyperactivity disorder (ADHD) in Japanese children with higher genetic susceptibility to ADHD with a birth weight under 2000 g
Source: BMC Med. 2021 Sep 24;19:229. doi: 10.1186/s12916-021-02093-3 (PMC8461893; doi:10.1186/s12916-021-02093-3)
Supplement: Supplementary file 7 — Additional File 7. Table S4 - Comparison of background characteristics of children included in the analysis and those excluded from the analysis due missing information on ADHD-RS or PRS. [file 12916_2021_2093_MOESM7_ESM.docx]

**Additional File 7: Table S4** - Comparison of background characteristics of children included in the analysis and those excluded from the analysis due missing information on ADHD-RS or PRS

| **Characteristics** | **Samples with complete information (N=659)** |  | **Excluded sample** | | |
| --- | --- | --- | --- | --- | --- |
|  |  |  | **Due to missing ADHD (N=422)** |  | **Due to missing PRS (N=137)** |
| *Children’s characteristics* |  |  |  |  |  |
| Birth order, n (%) |  |  |  |  |  |
| First-born | 329 (49.9%) |  | 219 (51.9%) |  | 66 (48.2%) |
| Gender, n (%) |  |  |  |  |  |
| Female | 324 (49.2%) |  | 202 (47.9%) |  | 68 (49.6%) |
| Gestational age at birth in week, mean (SD) | 39.99 (1.52) |  | 39.1 (1.47) |  | 38.9 (1.56) |
| Preterm birth n (%) | 42 (6.4%) |  | 18 (4.3%) |  | 9 (6.6%) |
| Birth weight |  |  |  |  |  |
| <2000 g | 16 (2.4%) |  | 5 (1.2%) |  | 3 (2.2%) |
| 2000-2499 g | 63 (9.6%) |  | 31 (7.4%) |  | 19 (13.9%) |
| >2500 g | 580 (88.0%) |  | 386 (91.5%) |  | 115 (83.9%) |
| *Parent’s characteristics* |  |  |  |  |  |
| Mother’s age at delivery, n (%) |  |  |  |  |  |
| <35 years | 477 (72.4%) |  | 339 (80.3%) |  | 90 (65.7%) |
| Maternal educational attainment, n (%) |  |  |  |  |  |
| ≤12 years | 208 (31.6%) |  | 168 (39.8%) |  | 38 (27.7%) |
| Maternal pre-pregnancy BMI, n (%) |  |  |  |  |  |
| Underweight (<18.5) | 148 (22.5%) |  | 82 (19.4%) |  | 22 (16.1%) |
| Normal weight (18.5 – 24.9) | 439 (66.6%) |  | 296 (70.1%) |  | 102 (74.4%) |
| Overweight (≥25.0) | 72 (10.9%) |  | 44 (10.4%) |  | 13 (9.5%) |
| Pre-pregnancy or during pregnancy smoking history, n (%) |  |  |  |  |  |
| Yes | 134 (20.3%) |  | 108 (25.6%) |  | 21 (15.3%) |
| Alcohol consumption during pregnancy, n (%) |  |  |  |  |  |
| Yes | 87 (13.2%) |  | 47 (11.1%) |  | 23 (16.8%) |
| Father’s age at birth |  |  |  |  |  |
| ≥35 years | 238 (36.1%) |  | 129 (30.6%) |  | 57 (41.6%) |
| Father’s education at birth |  |  |  |  |  |
| ≤12 years | 47 (7.1%) |  | 46 (10.9%) |  | 12 (8.8%) |
| Household annual income at birth in JPY |  |  |  |  |  |
| ≤ 3 million | 39 (9.2%) |  | 8 (5.8%) |  | 28 (4.3%) |
| 3-8 million | 314 (74.4%) |  | 104 (75.9%) |  | 500 (75.9%) |
| ≥ 8 million | 69 (15.4%) |  | 25 (18.3%) |  | 131 (19.9%) |

Note: ADHD, attention deficit/hyperactivity disorder; BMI, body mass index; IQR, interquartile range; PRS, polygenic risk score; SD, standard deviation; JPY, Japanese yen.
