# Supplementary material for: Elevated risk of attention deficit hyperactivity disorder (ADHD) in Japanese children with higher genetic susceptibility to ADHD with a birth weight under 2000 g
Source: BMC Med. 2021 Sep 24;19:229. doi: 10.1186/s12916-021-02093-3 (PMC8461893; doi:10.1186/s12916-021-02093-3)
Supplement: Supplementary file 8 — Additional File 8. Table S5 - Little’s test for Missing Completely at random and test for covariate-dependent missingness. [file 12916_2021_2093_MOESM8_ESM.docx]

**Additional File 8: Table S5** - Little’s test for Missing Completely at random and test for covariate-dependent missingness

| **Models**^†^ | **Covariates** | **No. of observation** | **Chi-square distance** | **Degrees of freedom** | **P value** |
| --- | --- | --- | --- | --- | --- |
| Model 1 | No covariates | 908 | 0.2809 | 2 | 0.8690 |
| Model 2 | Sex of child | 908 | 1.9106 | 4 | 0.7522 |
| Model 3 | Sex and birth order of child | 908 | 4.0455 | 6 | 0.6705 |
| Model 4 | Sex and birth order of child, birth weight | 908 | 4.4835 | 10 | 0.9229 |
| Model 5 | Sex and birth order of child, mother’s age at birth | 908 | 4.8440 | 8 | 0.7741 |
| Model 6 | Sex and birth order of child, mother’s age and education at birth | 908 | 5.4510 | 10 | 0.8591 |
| Model 7 | Sex and birth order of child, mother’s age and education at birth, pre-pregnancy smoking status | 908 | 7.2323 | 10 | 0.8419 |
| Model 8 | Sex and birth order of child, mother’s age and education at birth, pre-pregnancy smoking status and father’s age at birth | 908 | 14.5773 | 16 | 0.5558 |
| Model 9 | Sex and birth order of child, mother’s age and education at birth, pre-pregnancy smoking status, father’s age and education at birth | 908 | 14.0050 | 18 | 0.7288 |
| Model 10 | Sex and birth order of child, mother’s age and education at birth, pre-pregnancy smoking status, father’s age and education at birth, and household annual income | 908 | 14.0177 | 22 | 0.9008 |

Note: ^†^Models 2-10 were used to test the assumption of covariate-dependent missingness assumption.
