# Supplementary material for: Elevated risk of attention deficit hyperactivity disorder (ADHD) in Japanese children with higher genetic susceptibility to ADHD with a birth weight under 2000 g
Source: BMC Med. 2021 Sep 24;19:229. doi: 10.1186/s12916-021-02093-3 (PMC8461893; doi:10.1186/s12916-021-02093-3)
Supplement: Supplementary file 9 — Additional File 9. Table S6 - Association of birth weight categories and genetic risk with ADHD total score among Japanese children at age 8-9 years after multiple imputation of missing polygenic risk score for 137 children. [file 12916_2021_2093_MOESM9_ESM.docx]

**Additional File 9: Table S6** - Association of birth weight categories and genetic risk with ADHD total score among Japanese children at age 8-9 years after multiple imputation of missing polygenic risk score for 137 children (N=796)

| **Birth weight & genetic risk of ADHD** | **Rate Ratio (95% Confidence Intervals)** | | |
| --- | --- | --- | --- |
|  | **Model 1**^†^ | **Model 2**^‡^ | **Model 3**^§^ |
| Normal birth weight |  |  |  |
| Low risk (ref.) | 1.00 | 1.00 | 1.00 |
| High risk | 0.99 (0.84-1.18) | 0.98 (0.84-1.15) | 0.98 (0.84-1.15) |
| Birth weight: 2000-2499 g |  |  |  |
| Low risk | 0.90 (0.62-1.31) | 1.07 (0.73-1.56) | 1.06 (0.73-1.56) |
| High risk | 0.85 (0.59-1.22) | 0.92 (0.63-1.35) | 0.92 (0.63-1.35) |
| Birth weight <2000 g |  |  |  |
| Low risk | 1.36 (0.89-2.08) | 1.41 (0.88-2.25) | 1.38 (0.85-2.23) |
| High risk | **1.79 (1.14-2.80)*** | **1.72 (1.20-2.46)**** | **1.70 (1.18-2.44)**** |

Note: Normal birth weight was defined as birth weight ≥ 2500 g; ref., reference category; Values in bold show statistical significance; ** p<0.01; * p<0.05;

^†^Model 1 was adjusted for variations in survey time only;

^‡^Model 2 additionally adjusted for gender of child, parity, maternal age, education, pre-pregnancy body mass index, pre-pregnancy smoking status, alcohol intake;

^§^Model 3 additionally adjusted for father’s age at birth and household annual income.
