# Supplementary material for: Elevated risk of attention deficit hyperactivity disorder (ADHD) in Japanese children with higher genetic susceptibility to ADHD with a birth weight under 2000 g
Source: BMC Med. 2021 Sep 24;19:229. doi: 10.1186/s12916-021-02093-3 (PMC8461893; doi:10.1186/s12916-021-02093-3)
Supplement: Supplementary file 10 — Additional File 10. Table S7 - Association of birth weight categories and genetic risk with inattention and hyperactivity scores among Japanese children at age 8-9 years after multiple imputation of missing polygenic risk score for 137 children. [file 12916_2021_2093_MOESM10_ESM.docx]

**Additional File 10: Table S7** - Association of birth weight categories and genetic risk with inattention and hyperactivity scores among Japanese children at age 8-9 years after multiple imputation of missing polygenic risk score for 137 children (N=796)

| **Birth weight & genetic risk of ADHD** | **Rate Ratio (95% Confidence Intervals)** | | |
| --- | --- | --- | --- |
|  | **Model 1**^†^ | **Model 2**^‡^ | **Model 3**^§^ |
| **Inattention symptoms** |  |  |  |
| Normal birth weight |  |  |  |
| Low risk (ref.) | 1.00 | 1.00 | 1.00 |
| High risk | 0.97 (0.82-1.15) | 0.96 (0.82-1.13) | 0.97 (0.82-1.13) |
| Birth weight: 2000-2499 g |  |  |  |
| Low risk | 0.89 (0.61-1.28) | 1.01 (0.70-1.46) | 1.01 (0.70-1.45) |
| High risk | 0.89 (0.62-1.26) | 0.94 (0.65-1.38) | 0.95 (0.65-1.39) |
| Birth weight <2000 g |  |  |  |
| Low risk | 1.25 (0.89-1.75) | 1.30 (0.91-1.86) | 1.27 (0.87-1.86) |
| High risk | **1.67 (1.06-2.63)*** | **1.62 (1.14-2.31)**** | **1.62 (1.13-2.31)**** |
| **Hyperactivity symptoms** |  |  |  |
| Normal birth weight |  |  |  |
| Low risk (ref.) | 1.00 | 1.00 | 1.00 |
| High risk | 1.03 (0.83-1.28) | 1.04 (0.85-1.26) | 1.03 (0.85-1.25) |
| Birth weight: 2000-2499 g |  |  |  |
| Low risk | 0.92 (0.57-1.48) | 1.21 (0.71-2.06) | 1.20 (0.71-2.04) |
| High risk | 0.77 (0.46-1.28) | 0.84 (0.48-1.45) | 0.84 (0.49-1.44) |
| Birth weight <2000 g |  |  |  |
| Low risk | 1.61 (0.80-3.23) | 1.70 (0.81-3.58) | 1.64 (0.79-3.44) |
| High risk | **2.04 (1.14-3.64)*** | **2.00 (1.20-3.34)**** | **1.92 (1.15-3.21)*** |

Note: Normal birth weight was defined as birth weight ≥ 2500 g; ref., reference category; Values in bold show statistical significance; ** p<0.01; * p<0.05;

^†^Model 1 was adjusted for variations in survey time only;

^‡^Model 2 additionally adjusted for gender of child, parity, maternal age, education, pre-pregnancy body mass index, pre-pregnancy smoking status, alcohol intake;

^§^Model 3 additionally adjusted for father’s age at birth and household annual income.
