# Supplementary material for: Elevated risk of attention deficit hyperactivity disorder (ADHD) in Japanese children with higher genetic susceptibility to ADHD with a birth weight under 2000 g
Source: BMC Med. 2021 Sep 24;19:229. doi: 10.1186/s12916-021-02093-3 (PMC8461893; doi:10.1186/s12916-021-02093-3)
Supplement: Supplementary file 11 — Additional File 11. Table S8 - Sensitivity analysis for the association of birth weight categories and genetic risk with ADHD total score among Japanese children at age 8-9 years after recoding missing PRS to the end of the spectrum. [file 12916_2021_2093_MOESM11_ESM.docx]

**Additional File 11: Table S8** - Sensitivity analysis for the association of birth weight categories and genetic risk with ADHD total score among Japanese children at age 8-9 years after recoding missing PRS to the end of the spectrum

| **Birth weight & genetic risk of ADHD** | **Rate Ratio (95% Confidence Interval)^1^** | | | |
| --- | --- | --- | --- | --- |
|  | **Recode missing as high risk (N=796)^2^** | **Recode missing as low risk (N=796)^3^** | **Multiple Imputation (N=796)^4^** | **Analysis with complete information (N=659)^4^** |
| Normal birth weight |  |  |  |  |
| Low risk (ref.) | 1.00 | 1.00 | 1.00 | 1.00 |
| High risk | 0.98 (0.85-1.14) | 0.97 (0.83-1.12) | 0.98 (0.84-1.15) | 0.98 (0.83-1.15) |
| Birth weight: 2000-2499 g |  |  |  |  |
| Low risk | 1.02 (0.66-1.57) | 1.06 (0.77-1.45) | 1.06 (0.73-1.56) | 1.04 (0.68-1.61) |
| High risk | 0.97 (0.70-1.35) | 0.88 (0.56-1.37) | 0.92 (0.63-1.35) | 0.87 (0.56-1.37) |
| Birth weight <2000 g |  |  |  |  |
| Low risk | 1.30 (0.80-2.11) | 1.44 (0.95-2.18) | 1.38 (0.85-2.23) | 1.30 (0.79-2.16) |
| High risk | **1.71 (1.24-2.36)**** | **1.67 (1.15-2.43)**** | **1.70 (1.18-2.44)**** | **1.64 (1.14-2.35)**** |

Note: Normal birth weight was defined as birth weight ≥ 2500 g; ref., reference category; Values in bold show statistical significance; ** p<0.01; * p<0.05;

^1^Model was adjusted for variations in survey time, gender of child, parity, maternal age, education, pre-pregnancy body mass index, pre-pregnancy smoking status, alcohol intake, father’s age at birth, and household annual income;

^2^Missing ADHD-PRS of 137 children were considered as high risk;

^3^Missing ADHD-PRS of 137 children were considered as low risk;

^4^Results presented in the last two columns are included here for comparison purposes. The details of those models are presented in Tables 3-4 and Tables S6-S7 respectively.
