# Supplementary material for: Elevated risk of attention deficit hyperactivity disorder (ADHD) in Japanese children with higher genetic susceptibility to ADHD with a birth weight under 2000 g
Source: BMC Med. 2021 Sep 24;19:229. doi: 10.1186/s12916-021-02093-3 (PMC8461893; doi:10.1186/s12916-021-02093-3)
Supplement: Supplementary file 12 — Additional File 12. Table S9 - Sensitivity analysis for the association of birth weight categories and genetic risk with inattention and hyperactivity scores among Japanese children at age 8-9 years after recoding missing PRS to the end of the spectrum. [file 12916_2021_2093_MOESM12_ESM.docx]

**Additional File 12: Table S9** - Sensitivity analysis for the association of birth weight categories and genetic risk with inattention and hyperactivity scores among Japanese children at age 8-9 years after recoding missing PRS to the end of the spectrum

| **Birth weight & genetic risk of ADHD** | **Rate Ratio (95% Confidence Interval)^1^** | | | |
| --- | --- | --- | --- | --- |
|  | **Recode missing as high risk (N=796)^2^** | **Recode missing as low risk (N=796)^3^** | **Multiple Imputation (N=796)^4^** | **Analysis with complete information (N=659)^4^** |
| ***Inattention symptoms*** |  |  |  |  |
| Normal birth weight |  |  |  |  |
| Low risk (ref.) | 1.00 | 1.00 | 1.00 | 1.00 |
| High risk | 0.96 (0.83-1.11) | 0.96 (0.83-1.11) | 0.97 (0.82-1.13) | 0.96 (0.82-1.13) |
| Birth weight: 2000-2499 g |  |  |  |  |
| Low risk | 0.99 (0.65-1.51) | 0.97 (0.71-1.34) | 1.01 (0.70-1.45) | 1.02 (0.67-1.56) |
| High risk | 0.96 (0.69-1.34) | 0.98 (0.63-1.52) | 0.95 (0.65-1.39) | 0.97 (0.62-1.50) |
| Birth weight <2000 g |  |  |  |  |
| Low risk | 1.19 (0.8-1.78) | 1.33 (0.96-1.84) | 1.27 (0.87-1.86) | 1.19 (0.78-1.81) |
| High risk | **1.61 (1.17-2.22)**** | **1.61 (1.09-2.38)*** | **1.62 (1.13-2.31)**** | **1.56 (1.07-2.27)*** |
| ***Hyperactivity symptoms*** |  |  |  |  |
| Normal birth weight |  |  |  |  |
| Low risk (ref.) | 1.00 | 1.00 | 1.00 | 1.00 |
| High risk | 1.06 (0.88-1.28) | 1 (0.83-1.21) | 1.03 (0.85-1.25) | 1.03 (0.84-1.27) |
| Birth weight: 2000-2499 g |  |  |  |  |
| Low risk | 1.07 (0.61-1.88) | 1.29 (0.86-1.94) | 1.20 (0.71-2.04) | 1.10 (0.63-1.91) |
| High risk | 1 (0.65-1.54) | 0.61 (0.35-1.05) | 0.84 (0.49-1.44) | 0.63 (0.37-1.08) |
| Birth weight <2000 g |  |  |  |  |
| Low risk | 1.58 (0.77-3.23) | 1.71 (0.9-3.23) | 1.64 (0.79-3.44) | 1.64 (0.77-3.48) |
| High risk | **2.00 (1.27-3.14)**** | **1.86 (1.12-3.08)*** | **1.92 (1.15-3.21)*** | **1.87 (1.14-3.06)*** |

Note: Normal birth weight was defined as birth weight ≥ 2500 g; ref., reference category; Values in bold show statistical significance; ** p<0.01; * p<0.05;

^1^Model was adjusted for variations in survey time, gender of child, parity, maternal age, education, pre-pregnancy body mass index, pre-pregnancy smoking status, alcohol intake, father’s age at birth, and household annual income;

^2^Missing ADHD-PRS of 137 children were considered as high risk;^3^Missing ADHD-PRS of 137 children were considered as low risk;

^4^Results presented in the last two columns are included here for comparison purposes. The details of those models are presented in Tables 3-4 and Tables S6-S7 respectively.
